# 69. A case of two autoimmune conditions

**DOI:** 10.1093/rap/rky034.032

**Published:** 2018-09-20

**Authors:** Khin Yein, Azeem Ahmed

**Affiliations:** Rheumatology, Great Western Hospitals NHS Trust, Swindon, UNITED KINGDOM

**Introduction:** We present a case of large vessel vasculitis (Takayasu arteritis) developed in a patient with long-standing ulcerative colitis. There is a less common association between these two organ-specific autoimmune conditions.

**Case description:** A 47 year old woman was referred to our rheumatology department with a six-month history of weight loss of 8kg, fatigue, poor appetite and raised inflammatory markers of CRP > 140 mg/L and ESR 100 mm/h. Her ulcerative colitis, diagnosed in 1996, was initially well controlled with azathioprine 150 mg and asacol 800 mg daily. She started to have flare up of her bowel symptoms in early 2016 and despite the additional use of prednisolone, salofalk suppositories and increased dose of azathioprine 200 mg daily, her colitis continued to be active. She had one episode of bilateral anterior uveitis. Her treatment was escalated to biologic therapy. She developed skin reaction to infliximab after the first infusion. Adalimumab (40 mg fortnightly injections for three months and 80 mg fortnightly for three months) was ineffective in controlling the colitis. Golimumab was also ineffective and she had side effects with aches and pain in her joints. She developed bilateral ankle swellings as an allergic type reactions after three doses of vedolizumab. Due to therapeutic difficulty, surgery had been considered at one point. Fortunately, her colitis settled down reasonably well with a course of prednisolone shortly before vedolizumab was commenced. A repeat colonoscopy in June 2017 showed mild to moderate inflammation in rectum and distal sigmoid. She was in clinical remission from her colitis, although her inflammatory markers were mildly but persistently raised indicating that her colitis was histologically active. She presented to our rheumatology department in March 2018 with constitutional symptoms and weight loss. She had chest pain and shortness of breath on minimal exertion. She had no rash, joint swellings or other symptoms of giant cell arteritis or limb claudication. There were no recent foreign travel or tuberculosis contact.On examination, blood pressure on the right arm was 132/70 mmHg and left 128/76 mmHg. Peripheral pulses were present. Her heart sounds were normal, chest was clear and abdominal was soft and not tender. Her inflammatory markers were high with CRP 145 mg/L and ESR 100 mm/h. ANA, ANCA, rheumatoid factor, anti CCP antibodies, ACE, HIV, hepatitis B, C and quantiFERON Gold were negative. She had anaemia. CT neck, chest, abdomen and pelvis showed aortitis. The CT PET showed the entire aorta and its branches were affected consistent with the diagnosis of large vessel vasculitis (Takayasu arteritis type 5). She was commenced on 60 mg of prednisolone along with bone protection medications. She was counselled and treated with six cycles of cyclophosphamide infusions. She responded very well to the treatments and regained weight. Inflammatory markers normalised and she managed to reduce the prednisolone dose gradually.

**Discussion:** Takayasu arteritis is a systemic chronic granulomatous inflammation affecting mainly aorta and its main branches. It has an annual incident of 2.6 cases per million population, more common in women under 50 years of age and most frequently occur in Asia countries. People with HLA B52 serotype have increased risk. It is important to establish the correct diagnosis and evaluate the extent and severity of the arterial involvement. Although angiography is regarded as a gold standard investigation, it does not give any information on the state of the arterial walls. Magnetic resonant angiogram, CT angiogram, CT PET (18F-FDG) and colour Doppler ultrasound are non-invasive and supplementary in obtaining accurate diagnosis. No single imaging modality provides all the information required to detect all the affected lesions, the state of the lumens (stenosed, occluded, aneurysmal) and the thickness of the walls. They are also useful in assessment of the disease activity as maintenance therapy is required to acheive long term remission. In our patient, only aortitis in thoracic aorta was shown on CT. Further imaging with PET CT (18F-FDG) showed increased uptake in the entire aorta and its branches (subclavian, right carotid, left pulmonary artery, coeliac axis and superior mesenteric artery). Conventional glucocorticoids (prednisolone 1mg/kg/day) and immunosuppressive agents can induce remission. In inadequately controlled, relapsed and refractory cases, biologic agents such as anti TNF-alpha, IL-6 inhibitor and rituximab are reported to be effective in controlling the disease activity. Endovascular interventions may require in occluded lesions for revascularisation. Our patient’s colitis failed to improve on biologic agents either due to the ineffectiveness of the drugs or the adverse reactions towards the drugs.

**Key Learning Points:** This case reminds us to have an increased awareness of the coexistence of these two autoimmune conditions and in patients with unexplained raised inflammatory markers and constitutional symptoms, extra-intestinal inflammatory causes should be investigated. It highlights the fact that CT scan alone is not enough in evaluation of the disease extent. In this case arteritis in smaller arteries were not shown in the CT images. MR angiogram, CT angiogram and CT PET are superior in assessing the extent and severity of the inflammation affecting the lumen and wall of the arteries.

**Disclosure: K. Yein:** None. **A. Ahmed:** None.

